# Novel Electrokinetic Microfluidic Detector for Evaluating Effectiveness of Microalgae Disinfection in Ship Ballast Water

**DOI:** 10.3390/ijms161025560

**Published:** 2015-10-26

**Authors:** Myint Myint Maw, Junsheng Wang, Fabo Li, Jinhu Jiang, Younan Song, Xinxiang Pan

**Affiliations:** 1 College of Marine Engineering, Dalian Maritime University, Dalian 116026, China; E-Mail: parahitamyint@dlmu.edu.cn; 2 College of Information and Science Technology, Dalian Maritime University, Dalian 116026, China; E-Mails: bfli001@dlmu.edu.cn (F.L.); jiangjinhu@dlmu.edu.cn (J.J.); songyounan@dlmu.edu.cn (Y.S.)

**Keywords:** electrokinetic flow, fluorescence detection, microalgae, microfluidic chip, ballast water treatment

## Abstract

Ship ballast water treatment methods face many technical challenges. The effectiveness of every treatment method usually is evaluated by using large scale equipment and a large volume of samples, which involves time-consuming, laborious, and complex operations. This paper reports the development of a novel, simple and fast platform of methodology in evaluating the efficiency and the best parameters for ballast water treatment systems, particularly in chemical disinfection. In this study, a microfluidic chip with six sample wells and a waste well was designed, where sample transportation was controlled by electrokinetic flow. The performance of this microfluidic platform was evaluated by detecting the disinfection of *Dunaliella salina* (*D. salina*) algae in ballast water treated by sodium hypochlorite (NaClO) solution. Light-induced chlorophyll fluorescence (LICF) intensity was used to determine the viability of microalgae cells in the system, which can be operated automatically with the dimension of the detector as small as 50 mm × 24 mm × 5 mm. The 40 µL volume of sample solution was used for each treatment condition test and the validity of detection can be accomplished within about five min. The results show that the viability of microalgae cells under different treatment conditions can be determined accurately and further optimal treatment conditions including concentrations of NaClO and treatment time can also be obtained. These results can provide accurate evaluation and optimal parameters for ballast water treatment methods.

## 1. Introduction

Most ships have to carry water as ballast, which is not only essential to normal navigation but vital to ships’ safety. Such water often contains nonindigenous organisms that will be released when the ship deballasts. The consequences caused by the introduction of harmful aquatic organisms and pathogens are of major concern; on entry into new environments these have been shown to cause extensive damage to local ecologies and have a severe impact on fisheries and vital economic activities. They can also pose a threat to human health and safety [1,2]. The Global Environmental Fund (GEF) has identified the introduction of invasive alien species and pathogens through ballast water as one of the four major marine hazards [3]. The International Convention for the Control and Management of Ships’ Ballast Water and Sediments in 2004 stipulated that ballast water that may be discharged by ships should meet desired standards. The phase-in of Regulation D-2 of Ballast Water Management, Ballast Water Performance Standard, D-2 occurs considering the construction date and ballast water capacity of vessels, but from 2016 onwards all vessels need to comply with it. The soon expected entry into force of the Ballast Water Management (BWM) Convention is an important driving force for ballast water treatment technology developments worldwide. The Convention is retroactive in implementation and requires ballast water treatment systems installation on existing ships [4,5]. Many ballast water treatment systems have been developed during the past decades [6,7,8,9,10]. Currently the chemical method is the most commonly used way of ballast water treatment and in order to ensure the lethal effect of a one-time injection of chemical reagent, high concentrations may be required. These produce additional unwanted byproducts and thus cause environmental pollution that may impact human health. To resolve these problems, the optimum effectiveness of chemical reagent concentration and treatment time should be evaluated before treating ballast water. However the effectiveness of every treatment method usually is evaluated by using large scale equipment and a large volume of samples, which always involves time-consuming, laborious, and complex operations. A simple and fast platform methodology of evaluating the efficient parameters for ballast water treatment systems is presented here to overcome to some degree these problems. The performance of this platform is based on the detecting viabilities of microorganisms with the use of a novel electrokinetically controlled microfluidic detector.

The application of microfluidic chips in the life sciences has led to a diversity of new research directions. Compared with traditional methods, microfluidic chip analysis is highly efficient, small in size, easy to integrate, and low in overall cost [11,12,13,14]. Making a new microfluidic chip can be completed within a short time and it provides accurate measurement of micro-biological samples [15,16,17]. Microfluidic devices offer the advantages of precise control over experimental conditions via custom designed chip architectures, parallelization, automation, and direct coupling to miniaturized downstream analysis platforms [18]. As the microfluidic chip occupies few square centimeters, it is economical in terms of space and able to complete more complex processes and specific tasks. It is also friendly to the operating environment and thus is conducive to the development of portable instruments for a variety of applications.

In the last decade, microvalves are most widely used to drive samples in microfluidic devices. In the microvalve control process, a conventional piezoelectric valve can produce a large driving force with a fast response but the diaphragm can only produce a small movement even if a high voltage was generated [19]. A thermal-driven micro-valve consumes a large amount of power and takes a long reaction time [20]. The phase transition valve has the advantages of a simple structure and less material but it can be used one-time; it cannot achieve repeated switching [21]. By applying electroosmotic flow theory and electrophoresis to control flow through on-off into microchannel, the control system is greatly simplified. It does not require any complex component and can operate easily at any time.

Approaches to detecting microalgae activity include using a light-induced chlorophyll fluorescence (LICF) detector. While the system is operated by the fluorescence excitation, a specific frequency in light produced by microalgae, and the intensity of fluorescence is directly dependent on microalgae chlorophyll content and can show the level of microalgae activity [22]. The process based on LICF detection of microalgae is simple, fast and shortens detection time than the use of the traditional detection methods such as optical microscopy processing, dye fluorescence microscopy, flow cytometry, molecular and biochemical methods. These traditional methods are operationally complex with complicated steps, long cycles and often have shortcomings in fluorescent dyes that are used [23,24,25].

Ballast water brought by foreign ships accounts for a large proportion of microalgae spread. Microalgae are literally invading the world and causing huge economic and environmental damage [26,27,28]. Therefore, microalgae are the main target for treatment and detection in our experiment and *Dunaliella salina* (*D. salina*) algae have been used as sample of microalgae.

The viabilities of invasive microorganisms such as *D. salina* were detected after treatment with different concentrations of sodium hypochlorite solution (NaClO). Also, the same reagent concentrations were alternately introduced with different time durations. The manipulation of these conditions provides an accurate determination of the effective reagent concentration level and the optimum treatment time.

## 2. Results and Discussion

According to the two experiment sets, the experiments verified the system’s feasibility and reflect the advantages of the research contained in this paper. The generating laser power, temperature, system components and other experimental conditions were kept at the same setting during the whole experimental processing.

The amplitude of signals, which were displayed on the computer screen with Labview software, was directly proportional to the activity of the microalgae cells. While the larger the activity, the stronger corresponding signal variation. A single pulse signal represents the activity of each corresponding *D. salina* which flows through the detection zone. After subsequent experimental data processed for the two sets of the experimental program, an appropriate number of signal amplitudes were selected to calculate the average value of fluorescence signal. The number between 30 to 50 signals becomes the appropriate number and we set 40 for each group due to the test design. The relative activity of microalgae was the ratio of average value of the fluorescence signal amplitudes of each treated sample solution to the average value of signal amplitudes of ordinary sample solution under the same conditions.

(1) $$\text{Relative activity of microalgae}=\frac{\text{average value of signal amplitudes of treated sample soltuion}}{\text{average value of signal amplitude of (N1)ordinary sample solution}}$$

### 2.1. Relative Activity after Different Treatment Time

**Figure 1:**
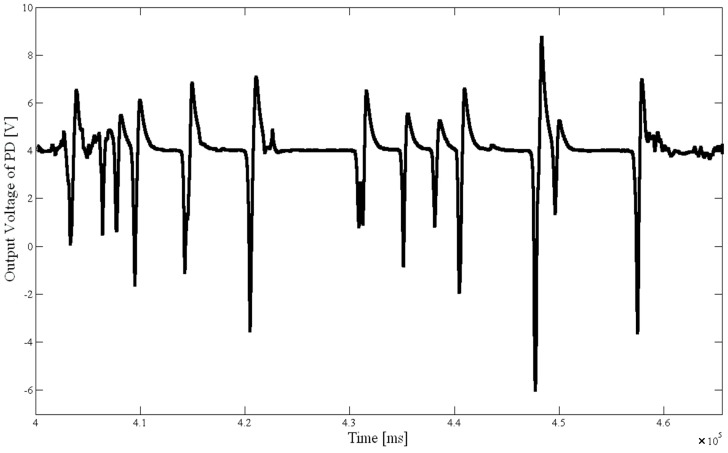
Fluorescence signal of *D. salina* after 6 min treatment with 3 mg/L of NaClO.

**Figure 2:**
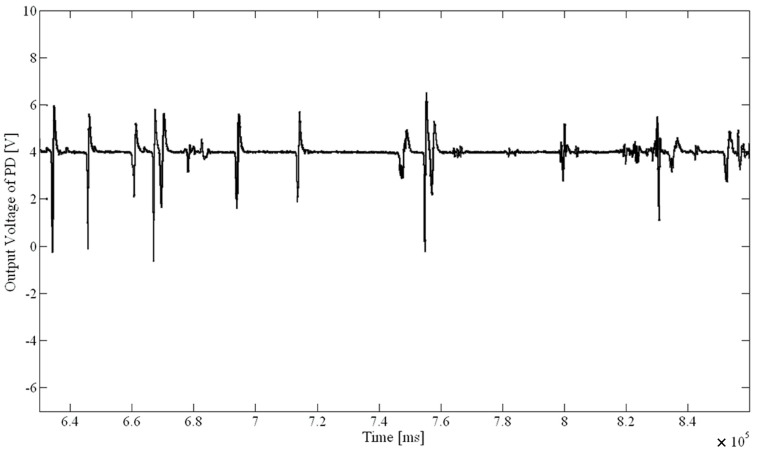
Fluorescence signal of algae when treated approximately 12 min with 3 mg/L of NaClO.

The following two figures described the charts of fluorescence signal results from set (II) experiments. These figures compared *D. salina* activities under different treatment times with sodium hypochlorite solution. The chart describes the output voltage varies with the treatment time. Figure 1 was the fluorescence signal diagram after 6 min treated with 3 mg/L sodium hypochlorite solution. Figure 2 was the fluorescence signal diagram for treatment about 12 min with 3 mg/L sodium hypochlorite solution.

The results of the two experiments were compared according to Figure 1 and Figure 2. The fluorescence signal for the treatment time of about 12 min was significantly smaller than the signal amplitudes with a treatment time of 6 min; the microalgae activity was clearly lowered. It shows the system is feasible and reliable by verifying the fact that chemical disinfection reduced the microalgae activity in a certain time.

### 2.2. Effect on D. salina through Treatment with Different Concentrations of NaClO at the Same Time

Experiment set (I) detected the activity of *D. salina* treated with different concentrations of sodium hypochlorite solution under the same duration. The treatment time was set at about 5 min. The power supply was converted into the required voltage value to control the microalgae at a desired sample well which could flow in the microchannel until it arrived at the detection zone while sample solutions from other sample wells were controlled to stop the flow. Nevertheless a small amount of sample solution from other undesired samples wells have also flown through the detection zone, but this had little effect on the results and so it can be ignored. After attaining the number of microalgae from the detection zone, the procedures for a next test sample solution were ready for the next detection. This in turn obtained fluorescence signals amplitude of five different groups.

**Figure 3:**
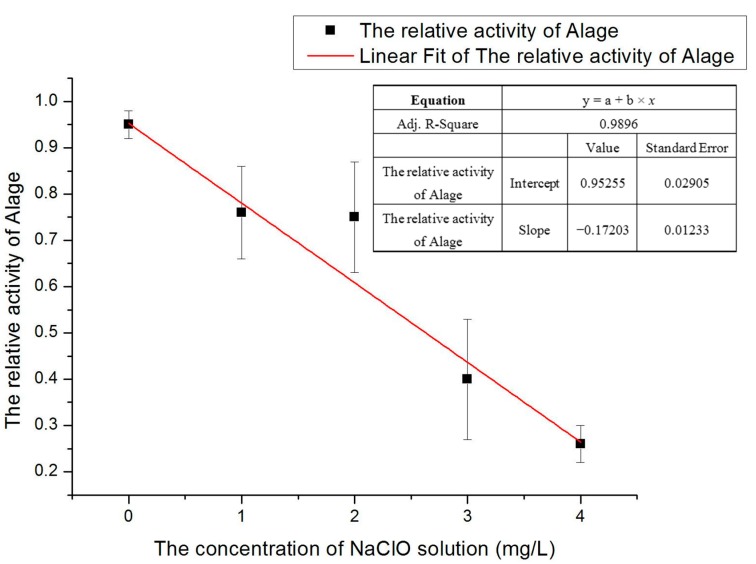
Graph of *D. salina* activity variation in accordance with the variation in concentrations of NaClO.

The purpose of data processing is as far as possible to avoid human errors, system errors or accidental factors in the experiment. To do data processing, the same number of fluorescence signals of the average value of peak voltages which represent the characterization of relative activity of microalgae were firstly selected. Selecting the appropriate fluorescent signal meant the signal which had already removed the abnormal pulse gross error, system generated error or outside interference. The time span for selecting signals in each group should not be too long. If the duration is long, it may affect the activity by sodium hypochlorite solution so that the accuracy of the results will be reduced. The graph of relative activity of microalgae versus with different concentration of sodium hypochlorite solution is shown in Figure 3. The chart clearly showed the activity of *D. salina* is dramatically decreased under increasing concentrations of sodium hypochlorite solution under the same treatment time.

### 2.3. Effect on D. salina Activity through Treatment at Constant Concentration of Sodium Hypochlorite Solution with Different Durations

**Figure 4:**
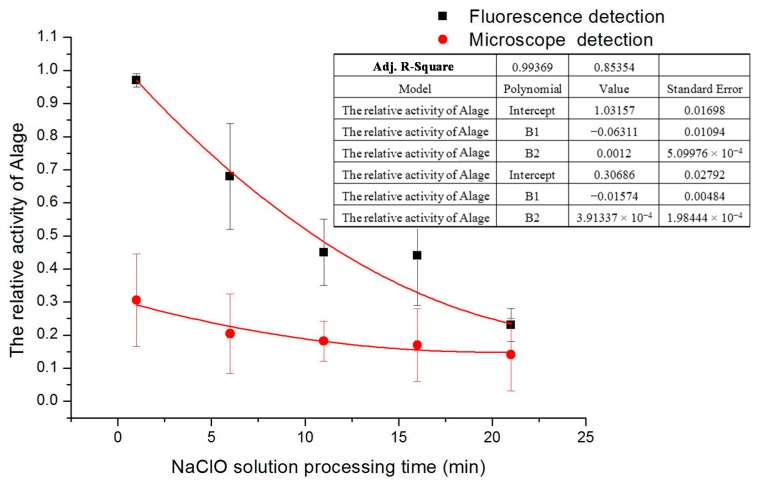
Relative activity of *D. salina* varies with time with 3 mg/L of NaClO treatment.

According to the experimental results of set (I), in Figure 3, if the concentration is too high it is difficult to observe the activity of the gradually changing processes. If the concentration is too small, the activity does not change significantly and thus does not facilitate the analysis of experimental results. Hence a moderate concentration of 3 mg/L sodium hypochlorite solution was chosen for the treatment of *D. salina*. During a certain time, the fluorescence signals amplitude was measured in the experiment. If the treatment time was too long, the signals magnitude would not be obvious. If the time was too short, it would be difficult to see the significant changes of signals amplitude. To avoid these two problems the treatment time was set at about 6 min. In this electrokinetic control method, there is a little delay time when implementing the experiment but it is never over 1 min. Likewise in data processing, there are some factors to be considered to remove gross error, systemic factors and outside interference signals that are generated. The statistic chart of the relative activity of microalgae varies with different treatment times of 3 mg/L concentration sodium hypochlorite solution under microscope detection and fluorescence detection is shown in Figure 4.

The relative activity of microalgae under microscope detection was calculated using a hemocytometer, (XB.K.25.0.10 mm, 1/400 mm^2^, Qiujing, China), to determine the concentration of microalgae cells in a sample solution. The treated sample solution was pipette into the plate and the cover glass placed on the sample. A 1:10 dilution was made with water. Cells were carefully counted on the five squares grid of surface plate and recorded. According to the number of cells counted in a square, area of the square, the height of the sample and dilution factor, total cells can be calculated by Equation (2).

(2) $$\text{Total cells}=\text{Total cells counted}\;\times \;\frac{\text{dilution factor}}{\text{no}.\text{ of squares}}\;\times \;10,000\;\text{cells}/\text{mL}\;\times \;\text{volume (mL)}$$

Counting and calculation was repeated three times for each test under the treatments of 6, 12, 18 and 24 min, respectively. Counting under the microscope and calculations could not be precise because it was very difficult to count moving cells. However both viable and non-viable unmoving cells were counted. The equation of relative activity of microalgae under microscope detection is as follow.

(3) $$\text{Relative activity of microalgae (mi)}=\frac{\text{Nct}}{\text{Ncc}}$$

where Ncc: Total cells of sample solution, Nct: Total living cells of sample solution treated with NaClO.

The final result from microscope detection shows that the living cells are decreasing with time (see Figure 4).

On the other hand, the relative activity of microalgae under fluorescence detection was calculated by applying Equation (1). It gives precise and clear data of microalgae activity on every minute while the detection under a microscope produces uncertain data. The main advantage of detection with fluorescence is that chlorophyll from even a dead cell can still be detected in practice [14]. Final results show that the increase in treatment time, the decrease *D. salina* activity and microalgae can be completely disinfected about 30 min by using 3 mg/L concentration of NaClO. Actually according to our experiments, when the relative activity of algae cells are more than 0.8, especially more than 0.85, the algae cells are very active and always move everywhere. However when the relative activity of algae cells is less than 0.2, especially less than 0.15, the algae cells hardly move in a long time and their activities are very low and close to dead. So, the threshold of relative activity should be within the range of from 0.15 to 0.2, above this threshold, algae cells are healthy.

## 3. Materials and Methods

### 3.1. The Detection System

#### 3.1.1. System Structure

**Figure 5:**
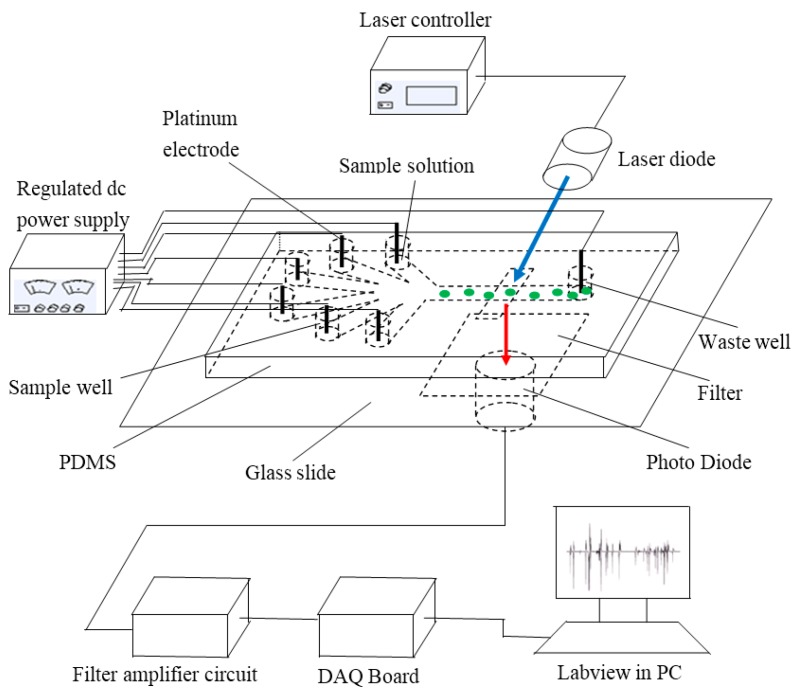
Diagram of system structure (the green dots represents the microalgae cells, the blue arrow represents the excitation light and the red arrow represents emitted chlorophyll fluorescence).

The system is mainly based on fluorescence detection using a microfluidic chip. This includes a signal detection sub-system, a signal processing sub-system, a data acquisition sub-system, a signal display sub-system and a direct current (DC) power supply module. The signal detection sub-system is composed of an electrode holder, a specific designed microfluidic chip, laser controller and laser diode (DL-488-050, the wavelength of 488 nm, Shanghai Xilong Opto electronics Technology Co., Ltd., Shanghai, China). The microchip was constructed by polydimethyl siloxane (PDMS) (Sylgard 184, Dow Corning, Midland, MI, USA) and a glass substrate (50 mm × 24 mm × 1.07 mm, CITOGLAS, Suzhou, China). It was designed with six sample wells at the left side and one waste well at the right side (see Figure 5). Platinum electrodes were inserted into six sample wells and the waste well. All these platinum electrodes were connected to low-voltage regulated DC power supply by the way of an electrode holder for the main purpose of controlling the flow of sample solution into the microchannel. In the signal processing part, there are three components: an emission filter (ET680, Chroma, Bellows Falls, VT, USA), photo diode PD (S8745-01 Hamamatsu, Bridgewater, NJ, USA), and filter amplifier circuit. An emission filter was mounted to filter fluorescence frequency between the microchip and a photo diode. To collect the data, data acquisition equipment NIusb-6259 board (National Instruments, Austin, TX, USA) was chosen. A personal computer (PC) with LabVIEW software (2011 version, National Instruments, Austin, TX, USA) finally displayed the signal results. All components were assembled as shown in Figure 5.

#### 3.1.2. Design and Fabrication of the Microfluidic Chip

**Figure 6:**
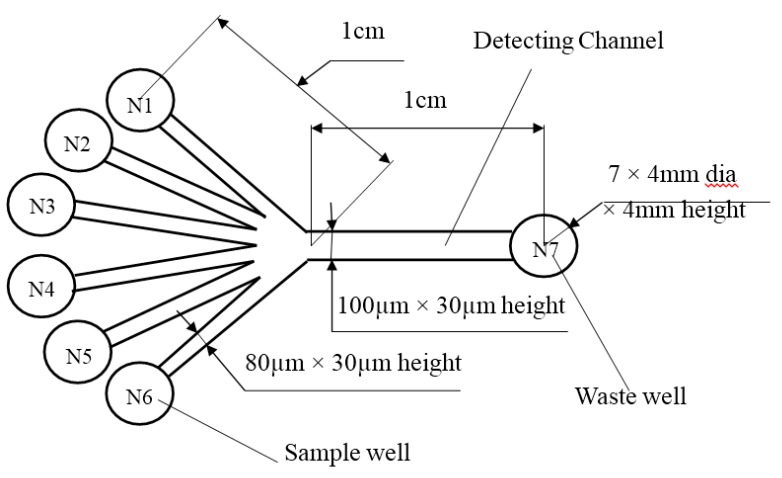
Structure and dimensions of a microfluidic chip.

A detailed design of the microfluidic chip and its dimensions are shown in Figure 6. The chip was fabricated by bonding a PDMS layer and a glass substrate through applying the soft lithography technology [29,30]. According to protocol for conducting experiments, the platform design contained six sample wells N1 to N6 with the dimension of 4 mm diameter × 4 mm height, and a waste well N7. The seven wells were formed by punching holes in the PDMS layer. Each of the sample wells is connected to the waste well by microchannels as shown in Figure 6. The detection zone was located at the center of the detection channel as indicated in Figure 6. The chip finally was cleaned by using plasma cleaner (HARRICK PLASMA). Afterward, the chip was carefully placed on the testing area to ensure the direction of laser incident rays was aligned with the detection zone. The laser diode was also set up with an appropriate distance to the chip.

### 3.2. Preparation of Sample Solution

#### 3.2.1. Culture of *Dunaliella Salina*

*Dunaliella salina* (*D. salina*), a kind of unicellular microalgae, was obtained from Liaoning Sea Fisheries Research Institute (LSFRI, Dalian, China). It was cultured in a 5000 mL specification Erlenmeyer flask with 3000 mL seawater medium that was enriched with a certain amount of trace element solution and vitamin solutions. After preparation of culture medium, it was immediately placed under incubation at room temperature.

#### 3.2.2. Titration of Sodium Hypochlorite Solution (NaClO)

At first the chlorine content in sodium hypochlorite solution was determined. NaClO was dissolved in seawater by setting the ratio 1:10. The chlorine level was determined by the iodine method; Secondly, required reagents for titration was systematically prepared by using 10% potassium iodide (KI) solution, 0.5% starch solution, 2 mol/L H_2_SO_4_ solution, 0.1 mol/L K_2_Cr_2_O_7_ standard solution and 0.1 mol/L Na_2_S_2_O_3_ solution; Thirdly Na_2_S_2_O_3_ solution was standardized in the dark and titrated until the color changed from blue to bright green; Finally, the volume of sodium hypochlorite and chlorine content was calculated. Sodium hypochlorite is very unstable, especially when there is rapid decomposition through light and heat, and therefore it is kept in the dark at a low temperature.

### 3.3. Working Principle

Firstly, test sample solutions which combined culture medium solution and titrated sodium hypochlorite solution were filled by a digital pipette into all wells; Secondly, electrodes were put into the wells and connected with the power supply. Low-voltage regulated DC power supply controlled the desired value of electrical potential to each electrode. This control causes the flow of sample from the sample well to the waste well by the way of specific micro-channel; Thirdly, by understanding the principle of microalgae fluorescence excitation and their spectrum, the laser controller was adjusted to fit the power. It is irradiated within a certain frequency to the detection zone of the chip. The laser diode was used as an excitation source. When the sample solution flows through the detection region under laser irradiation, the excited fluorescence can be observed. The emitted optical fluorescence was filtered by a red filter. Afterwards, the optical signal result of chlorophyll fluorescence was received by the photodiode and converted into electrical signals. The output voltage of the photodiode is proportional to the corresponding chlorophyll fluorescence intensity, that is, the signal amplitude objectively reflects the size and activity of microalgae. A differential amplifier circuit was designed to improve the signal-to-noise ratio of the output signal from the photodiode; Finally, data acquisition equipment (DAQ) board was used for acquiring and processing the signals from the amplifier circuit. DAQ collected the data and finally signal results were displayed by a computer with LabVIEW software. A displayed downward signal peak represents the fluorescence signal amplitude produced by living algae. The whole experiment was repeated three times.

### 3.4. Electrokinetic Control on a Microfludic Chip

To understand the electrokinetic phenomenon in microchannels, electroosmotic flow (EOF) and electrophoresis theory were applied along with the induced Joule heating effect. All these theories are based on electro hydrodynamic theory [19]. Our research involves the control of the flow involves the control of the on/off flow of sample solution in the microchannel by varying electric field intensities and we therefore applied the above-mentioned theories in our experiments.

#### 3.4.1. Analysis of Control by Electroosmosis

Current research applications of electrical phenomena in microfluidic basically use a model proposed by Stern [31]. The main one is the electric-double-layer potential distribution theory based on the Poisson-Boltzmann equation [32], which is able to derive the specific electric-double-layer potential distribution. Although the Poisson-Boltzmann equation is limited by solving the non-linear conditions, this equation can be used to describe the state when the fluid is in the fully developed stage in the passage. According to the electric double layer theory, the microfluidic chip’s channel wall becomes polarized when in contact with an electrolyte solution [33]. The factors of electric property depend on the pH value of the solution, electrolyte concentration, ionic strength, the characteristic of channel inner surface. However electricity directly determines the quantity of positive/negative ions and the wall surface ζ potential. Due to the effect of ions, viscous force or solvent action, ion migrations of diffusion layer occurred [34,35]. These ions migrations will carry the liquid forming an electroosmotic flow. The Von Smoluchowski equation [36] gives the expression of electroosmotic flow velocity:

(4) $$u_{\mathrm{eo}}=-\frac{{\text{εε}}_{0}\text{ζ}}{\text{μ}}E$$

where *u*_eo_ is the electroosmotic velocity, ε is relative dielectric constant of the electrolyte solution, ε_0_ is the vacuum dielectric constant, μ is fluid viscosity , ζ is the ζ potential at the wall surface, and *E* is electric field intensity. It can be seen that the electric field intensity, fluid viscosity and wall ζ potential all affect the velocity of electroosmotic flow. From the application point of view, the control of electroosmotic flow can be achieved by controlling the wall ζ potential, dielectric properties of the solution and external electric field.

#### 3.4.2. Analysis of Control by Electrophoresis

Electrophoresis is the motion of a charged particle under the effect of the electric field [37] that is, the electric field in the electrolyte solution and its force toward the electrode of opposite moving phenomenon. In an electrolyte solution, a charged particle experiences the Coulomb’s force and the viscous drag force. When these two forces are balanced, the charged particle achieves a steady moving speed, namely electrophoretic velocity *u_ep_*. The electrophoretic velocity of spherical particles is given by:

(5) $$u_{ep}=\frac{{\text{εε}}_{0}{\text{ζ}}_{\text{p}}}{\text{μ}}E$$

where *u_ep_* is electrophoretic velocity, and ζ_p_ is the ζ potential of the particle surface.

#### 3.4.3. Analysis of Control by Electrokinetics on a Microfluidic Chip

It is clearly observed in the above equations that the kinematic velocity of the charged particles in an electrostatic field is related to the external electric field intensity, the characteristic of electrolyte solution, and the surface charges of the wall and particles. The vector sum of electroosmotic flow velocity and electrophoretic velocity becomes the kinematic velocity of the charged particles in the electrolyte [38].

(6) $$up\;=\;\frac{{\text{εε}}_{0}}{\text{μ}}\left(-\text{ζ}+{\text{ζ}}_{\text{p}}\right)E$$

In the above Equations (2) and (3), the velocity of charged particles in the electrolyte solution is closely related with ζ potential of the particle surface ζ_p_ and ζ potential of the channel wall surface ζ while dynamic viscosity μ, electric field intensity *E*, relative dielectric constant of the medium ε, and permittivity of vacuum ε_0_ are unchanged. Due to this equation, the following conclusion was reached. When the two ζ potentials have opposite signs: it means one ζ potential is positively charged and another one is negatively charged; the directions of electroosmotic velocity and electrophoresis velocity will be the same. When the two ζ potential parameters are represented by the same symbol, the electrophoretic velocity direction will be opposite to the direction of electroosmotic velocity in the micro-channel. The actual speed of the electrokinetic particle velocity in the micro-channel is the vector sum of electrophoretic velocity and electroosmotic velocity.

After the power switch is turned on, *D. salina* flows in the direction from the negative pole to the positive pole. According to the simulation results and experimental phenomena software point of view, the flow effect of sample solution will be more distinct when applied to zero voltage at the inlet and applied 80–100 voltage at the outlet. Generally *D. salina* will not flow or flow slightly in the micro-channel when 40–50 voltage is applied at the inlet. So, we adopted three optimization types of voltages, 90, 45 and 0 V. This is dictated by the power switch on/off to control flow through into microchannels by electricity instead of using the traditional role of micro-valve control.

### 3.5. Experimental Procedures

Two main detection sets were researched in our experiment. A first set was the detection of *D. salina* activity after treatment with the use of different concentrations of sodium hypochlorite solution under the same processing time. Treatment time was set at about five min. The second was detection by using the same concentration of sodium hypochlorite solution in different treatment time spans, which is in accordance with a certain time gradient detection of its activity. According to the results in the first set, 3 mg/L sodium hypochlorite solution was chosen as an effective of point of view. The deficiency of *D. salina* for every second can be observed during the whole processing time.

To start detection for the first set, 30 µL of ordinary culture solution was first loaded by pipette into each well and then 10 µL titrated sodium hypochlorite solution with varying levels of concentrations which set at 0, 1, 2, 3 and 4 mg/L were respectively added into N2, N3, N4, N5 and N6 sample wells while a 10 µL culture solution was added to N1 and a 10 µL pure water into the waste well. When the treatment time for the sample solution was near to five min, the power supply was turned on to electrodes and also the laser power was switched on and adjusted. The electrode at the waste well was set to 90 V. The N1 sample solution was first allowed to flow in the microchannel. The situation of *D. salina* swimming in the microchannel could be observed under a microscope (Nikon Eclipse Ti-E, Nikon, Kobe, Japan). By applying LICF detector, PD and DAQ, detected *D. salina* algae signals were displayed on PC and data recorded. At a certain time after the first detection, electrode voltages were tuned to allow only the flow of N2 sample solution to the detection zone. The same procedure was then continued to detect *D. salina* activity that was treated with the concentrations of 1, 2, 3 and 4 mg/L sodium hypochlorite solution in order. After all the testing was completed, the data was saved and analysis was conducted.

In the second set of experiments, the detecting procedure was roughly the same as the first one; the main difference was the treatment time with the same sodium hypochlorite solution in the second set. A desired amount of 30 µL culture solution was loaded into all wells and another 10 µL of 3 mg/L concentration sodium hypochlorite solution was added into each of the sample wells except N1. Firstly, the culture solution in N1 flowed through the detection channel under the control of electric potential and the detected data results saved. After 5 min of treatment with NaClO, the sample solution in N2 was allowed to flow and also detected. After another 5 min, the sample solution in N3 flowed in the channel and was detected. Similarly the sample solutions in N4, N5 and N6 were tested under the same duration. After approximately 30 min, the whole detection process was completed. The data results were carefully saved in each step of detection.

## 4. Conclusions

In this study, a new microfluidic platform for fast evaluation of effectiveness of a ballast water chemical treatment method is presented. The novel features of the microfluidic platform described here include the following: (1) Effectiveness of a ballast water treatment method can be evaluated by the viability of microalgae cells under different chemical treatment conditions on a microfluidic chip; (2) The viability of microalgae cells was represented by average intensities of chlorophyll fluorescence emitted when the cells are passing through the detection zone in a microfluidic chip; (3) Sample transportation is controlled automatically by electrokinetic flow in a microfluidic chip, which is simple, easy to operate and small in size; (4) Compared with the traditional methods, the developed microfluidic system has some advantages such as small volume of sample solution required, speed, accuracy and low cost; and (5) It has great potential be extended to become a common evaluation platform in ship’s ballast water treatment systems as well as chemical treatment systems.
